# The Role of Coagulation and Complement Factors for Mast Cell Activation in the Pathogenesis of Chronic Spontaneous Urticaria

**DOI:** 10.3390/cells10071759

**Published:** 2021-07-12

**Authors:** Yuhki Yanase, Shunsuke Takahagi, Koichiro Ozawa, Michihiro Hide

**Affiliations:** 1 Department of Pharmacotherapy, Graduate School of Biomedical and Health Sciences, Hiroshima University, Hiroshima 734-8553, Japan; ozawak@hiroshima-u.ac.jp; 2 Department of Dermatology, Graduate School of Biomedical and Health Sciences, Hiroshima University, Hiroshima 734-8551, Japan; takshuns@gmail.com; 3 Department of Dermatology, Hiroshima City Hiroshima Citizens Hospital, Hiroshima 730-8518, Japan

**Keywords:** chronic spontaneous urticaria (CSU), mast cells, leukocytes, coagulation factors, endothelial cells, complement components, protease activated receptor (PAR), activated complement factor 5 (C5a)

## Abstract

Chronic spontaneous urticaria (CSU) is a common skin disorder characterized by an almost daily recurrence of wheal and flare with itch for more than 6 weeks, in association with the release of stored inflammatory mediators, such as histamine, from skin mast cells and/or peripheral basophils. The involvement of the extrinsic coagulation cascade triggered by tissue factor (TF) and complement factors, such as C3a and C5a, has been implied in the pathogenesis of CSU. However, it has been unclear how the TF-triggered coagulation pathway and complement factors induce the activation of skin mast cells and peripheral basophils in patients with CSU. In this review, we focus on the role of vascular endothelial cells, leukocytes, extrinsic coagulation factors and complement components on TF-induced activation of skin mast cells and peripheral basophils followed by the edema formation clinically recognized as urticaria. These findings suggest that medications targeting activated coagulation factors and/or complement components may represent new and effective treatments for patients with severe and refractory CSU.

## 1. Pathogenesis of Chronic Spontaneous Urticaria (CSU)

**Figure 1:**
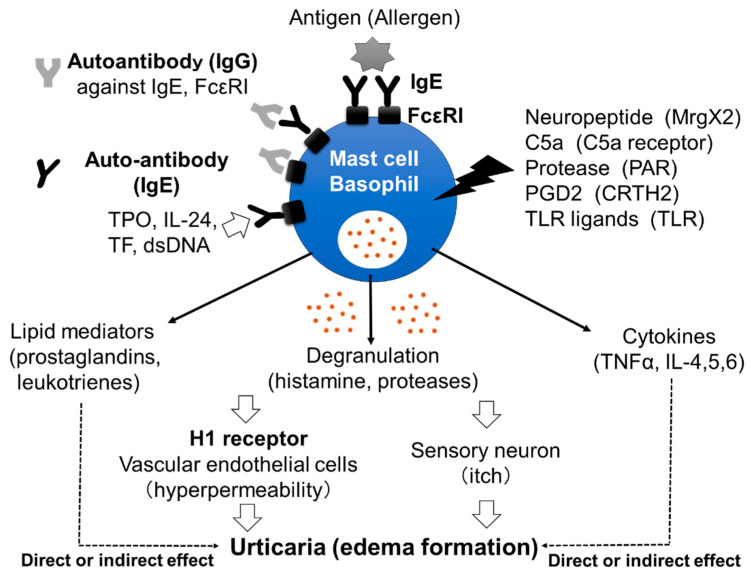
Mast cells’ and basophils’ activation by various stimuli. IgG autoantibodies against IgE antibodies and/or FcεRIs induce the activation of mast cells and basophils, followed by the release of stored mediators, such as histamine. Moreover, IgE autoantibodies against molecules, such as dsDNA, tissue factor TF, IL-24 and TPO, may also activate mast cells and basophils. Released histamine stimulates vascular endothelial cells and sensory neurons, resulting in edema formation with itch. Neuropeptides, complement components, proteases, prostaglandins and Toll-like receptor (TLR) ligands may also activate skin mast cells via corresponding receptors and induce the release of inflammatory mediators. Late-phase release of lipid mediators and cytokines may contribute to the exacerbation of CSU.

Chronic spontaneous urticaria (CSU), also called chronic idiopathic urticaria (CIU), is a skin disorder characterized by daily or almost daily recurring skin edema and flare with itch and pruritus anywhere on the body for more than 6 weeks [1]. The effectiveness of H1 anti-histamines for the treatment of CSU proves the important role played in the disorder by histamine, released from skin mast cells and/or peripheral basophils, and the histamine H1 receptor (H1R) (Figure 1) [2,3]. However, the exact mechanism by which mast cells and basophils are activated in the skin of patients with CSU remains unclear. Several reports have suggested that around 30–50% of patients with CSU have IgG autoantibodies against IgE antibodies and/or the high-affinity IgE receptors (FcεRIs) expressed on mast cells and basophils, which induce the activation of the cells followed by the release of stored mediators, such as histamine (Figure 1) [4,5]. Moreover, IgE autoantibodies against several molecules, such as dsDNA, tissue factor (TF), interleukin (IL)-24 and thyroid peroxidase (TPO), have also been detected in certain populations of patients with CSU (Figure 1) [6,7,8,9]. Shefler et al. reported that IL-24 is released from human mast cells in response to microvesicles derived from activated T cells, suggesting the contribution of IL-24 and anti-IL-24-IgE to the activation of mast cells and basophils in CSU [10]. They also induce the production and release of lipid mediators, such as leukotrienes, and cytokines from mast cells and/or basophils, which may contribute to the pathogenesis of CSU (Figure 1). A role of IgG autoantibodies against IgE antibodies and IgE autoantibodies against self-molecules in CSU has been suggested by the effectiveness of omalizumab, a therapeutic anti-IgE monoclonal antibody [11]. Infections by microorganisms, such as bacteria and/or virus, have also been suggested as exacerbating factors in CSU [12,13]. However, details of how infections are involved in the pathogenesis of CSU remain unclear. An increase in the levels of several internal molecules, such as D-dimer, fibrin/fibrinogen degradation products (FDP), prothrombin fragment 1+2 (PF_1+2_), C-reactive protein (CRP), substance P (SP) and pro-inflammatory cytokines, such as tumor necrosis factor (TNF)α, transforming growth factor (TGF)β, IL-1β, IL-6, IL-17, IL-31 and IL-33, has been reported in the blood of patients with CSU [14,15,16]. Several kinds of neuropeptides, such as SP, also activate mast cells, but not basophils, via Mas-related G protein-coupled receptor X2 (MrgX2), resulting in the release of histamine. Moreover, upregulation of MrgX2 expression on the skin mast cells of patients with severe CSU has been reported, suggesting that neuropeptides may also contribute to the activation of mast cells via MrgX2 [17]. On the other hand, plasma levels of IL-35 and vitamin D in patients with CSU were decreased [14]. Therefore, the activation of the IL-35- and/or vitamin D-related pathway may be an effective target for the treatment of CSU. In addition, various kinds of molecules which target cytokines and receptors, such as IL-4, IL-5, IL-13, TSLP, C5a, C5a receptor (C5aR) and Chemoattractant receptor-homologous molecule on Th2 cells (CRTH2), are being investigated/studied in clinical trials as medicines for CSU, suggesting that the pathogenesis of CSU is considerably more complicated than previously understood [18].

## 2. The Role of the Coagulation System

**Figure 2:**
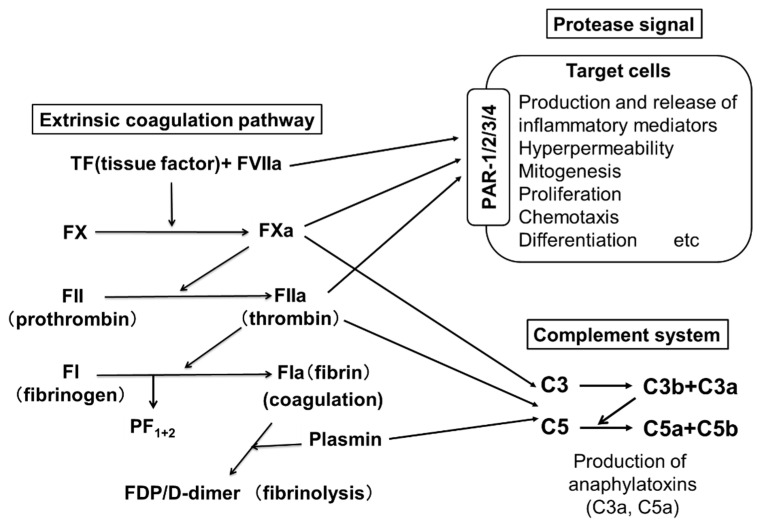
The extrinsic coagulation pathway and activation of PARs and complements by active form of coagulation factors. PARs, C3aR and C5aR are expressed on the surfaces of skin mast cells and peripheral basophils. Since FVIIa, FXa, FIIa and plasmin are serine proteases, FVIIa activates target cells via PAR-2. FXa and FIIa activate various types of cells via PAR-1,2,3 and PAR 1,3,4, respectively. Fibrin is also able to promote an inflammatory response via Toll-like receptor (TLR)-4. Moreover, activated coagulation factors (FXa and FIIa) produce C5a and C5b from C5, and C3a and C3b from C3.

Two pathways are involved in blood coagulation: the intrinsic and the extrinsic coagulation pathways. An important role for the extrinsic coagulation system triggered by TF and activated coagulation factors has been suggested in the pathogenesis of CSU. Figure 2 shows the pathway of the extrinsic coagulation. A few reports have suggested an improvement of CSU upon treatment with heparin, an activator of anti-thrombin, which inactivates thrombin (FIIa) [19,20]. Warfarin, which inhibits the production of coagulation factors, such as FVII, FX, FIX and FII, has also been suggested to be effective for CSU [21]. These findings imply that the activation of the extrinsic coagulation cascade and subsequently activated factors plays an important role as the trigger of CSU. PF_1+2_ is generated when FXa changes prothrombin (FII) to thrombin (FIIa), followed by the generation of fibrin (FIa) polymers (coagulation step) from fibrinogen (FI). D-dimer and FDP are generated during the degradation process of fibrin polymers by plasmin (fibrinolysis step) (Figure 2). Asero et al. and our group reported that plasma levels of PF_1+2_, FDP and D-dimer in patients with CSU were proportional to the severity of CSU symptoms [22,23]. Moreover, the thrombin generation capability of the extrinsic coagulation cascade, which is triggered by TF and produces thrombin (FIIa) in the plasma of patients with CSU, has been enhanced compared to healthy donors [24]. When a small amount of FVIIa in plasma binds to TF expressed on the cell surface, the extrinsic coagulation cascade is activated together with phosphatidylserine (PS) and Ca^2+^, and produces active forms of coagulation factors, such as FXa and FIIa. The FIIa then converts fibrinogen (FI) to fibrin (FIa), which forms fibrin polymers as a blood clot.

## 3. The Role of Protease-Activated Protein (PAR)

Protease-activated receptors (PARs) are known as a subfamily of G protein-coupled receptors that are activated by cleavage of their extracellular domain by specific serine proteases, such as trypsin, tryptase and active forms of coagulation factors, and mediate inflammation [25]. There are four mammalian PAR families (PAR-1/2/3/4). PARs are expressed on the surfaces of several cell types, such as platelets, endothelial cells, monocytes, mast cells and neurons [25]. Among the active forms of coagulation factors, FVIIa activates target cells via PAR-2, while FXa and FIIa activate PAR-1,2,3 and PAR-1,3,4, respectively [24]. Fibrin is also able to promote an inflammatory response via TLR-4 [25]. Several reports suggested that PAR-2 agonists, such as trypsin, activate human mast cells [26,27]. Moreover, the expression of PAR-2 on human mast cells is upregulated in urticarial lesions compared with non-urticarial lesions [28]. We also detected clear expression of PAR-1 and marginal expression of PAR-2 mRNA in human skin mast cells [29]. Nevertheless, we could not detect histamine release from skin mast cells in response to TRAP-6, a PAR-1 agonist, or AC55541, a PAR-2 agonist [29].

## 4. The Role of Vascular Endothelial Cells in CSU

**Figure 3:**
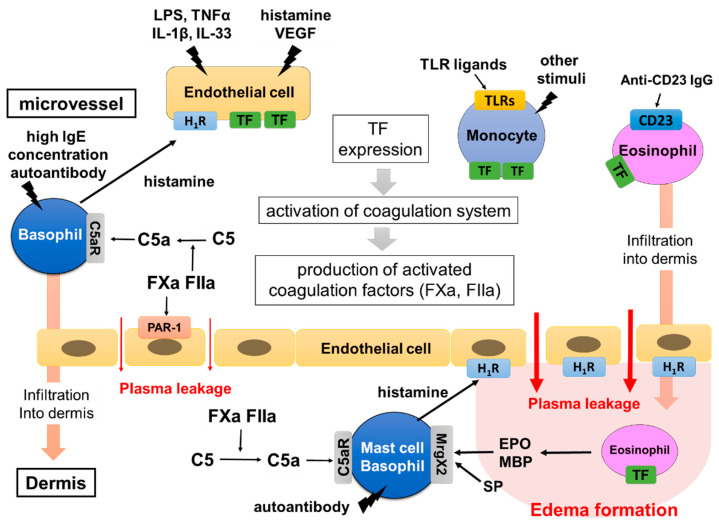
Hypothetical mechanism of edema formation triggered by TF in CSU. TF inducers, such as VEGF, TNF, IL-33, TLR ligands and anti-CD23 antibody, induce TF expression of vascular endothelial cells, monocytes and eosinophils. Histamine released from basophils in response to high IgE concentrations (>1 μM) may contribute to the synergistic expression of TF on vascular endothelial cells. The high expression of TF then activates the extrinsic coagulation system and produces active forms of the coagulation factors, such as FXa and FIIa. They induce plasma leakage via PAR-1 expressed on vascular endothelial cells and produce C5a from C5 in the intravessel and dermis. C5a leaks from blood vessels and is newly produced from plasma being exposed to eosinophils expressing TF in the tissue, then activating skin mast cells. Finally, skin mast cells release a large amount of histamine, which forms edema and flare via H1R. Basophils and monocytes stimulated with C5a, and eosinophils stimulated with anti-CD23 antibodies, may induce infiltration of the cells into the dermis and contribute to the exacerbation of edema formation.

In order to form edema and flare, which are clinically recognized as urticaria, plasma leakage from the skin microvessel into the dermis is a critical step. Generally, histamine released from skin mast cells and/or peripheral blood basophils is considered to be the major cause of plasma leakage from the skin microvessel, resulting in edema and flare. Vascular endothelial growth factor (VEGF), bradykinin and/or platelet active-activating factor (PAF) are also known to induce plasma leakage through gap formation between vascular endothelial cells via corresponding specific receptors [30]. However, the detailed role of vascular endothelial cells in plasma leakage, especially at local areas of the skin in CSU, remains unclear. Our in vitro studies, focused on the role of vascular endothelial cells, have unveiled a contribution of vascular endothelial cells in the early stage of CSU pathogenesis [31]. We have demonstrated that human umbilical vein endothelial cells (HUVECs) and human dermal microvascular endothelial cells (HMVECs) express a large amount of TF on their surface in response to the combination of several molecules (TF-inducers), such as histamine, VEGF, lipopolysaccharide (LPS), TNF-α, IL-33 and IL-1β [32]. The TF inducers are divided into two groups. Group 1 contains LPS, TNF-α, IL-33 and IL-1β, which activate the nuclear factor-kappa B (NF-κB)-related signaling pathway in vascular endothelial cells. Group 2 contains histamine and VEGF, which activate the phospholipase C-linked pathway. High expression of TF on vascular endothelial cells was induced synergistically by co-stimulation of two types of molecules, one in Group 1 and another in Group 2 TF inducers. Moreover, a large amount of TF expressed on HUVECs induced the activation of the extrinsic coagulation pathways and produced active forms of the coagulation factors, such as FXa and FIIa. Fxa and FIIa then induced gap-formation of vascular endothelial cells via PAR-1 and leakage of plasma out of blood vessels (Figure 2 and Figure 3) [31,32]. We also reported that adenosine, a metabolite of ATP, suppressed TF expression on vascular endothelial cells in response to the TF inducers, suggesting that adenosine-related molecules might be effective therapeutically for CSU [29]. Although clinical evidence on the role of TF expressed on vascular endothelial cells is still limited, further studies on the clinical roles of vascular endothelial cells would allow us to develop useful drugs and treatments for patients with severe and refractory CSU, targeting the TF-extrinsic coagulation pathway–plasma leakage axis.

## 5. The Role of Leukocytes in CSU

TF is normally observed in tissues outside blood vessels, but has also been reported as being expressed in blood cells, such as monocytes and eosinophils [33]. We recently reported that the expression of TF is high on the surfaces of peripheral blood monocytes of patients with CSU compared to healthy donors [34]. Moreover, we demonstrated that TF-expressing monocytes can trigger the extrinsic coagulation cascade and produce activated coagulation factors such as FXa and FIIa, followed by the induction of intercellular gap formation of HUVECs [34]. Furthermore, we clarified that several kinds of TLR ligands, such as LPS, TLR-4 agonist, and FLA-ST, TLR-5 agonist, induced TF expression of peripheral blood monocytes in vitro [34]. The involvement of chronic infections, especially due to Helicobacter pylori, has been mentioned as an underlying cause of CSU, but the underlying mechanism of mast cell activation by such infections remains unclear [12] Our results clarified that infections may contribute to the pathogenesis of CSU via the TF-triggered coagulation cascade. Consequently, TF expression on monocytes may be utilized as a marker for pathological conditions of CSU and a therapeutic target of severe and refractory CSU. Several papers have reported that C5a may recruit monocytes and prompt their infiltration of urticarial lesions. Thus, monocytes may cause further activation of coagulation factors that have leaked into the skin [35,36]. Moosbauer et al. revealed an increase in TF on the surfaces of eosinophils in response to several mediators and/or cytokines, such as platelet activating factor (PAF) and IL-5 [37]. Moreover, Cugno et al. showed TF expression on eosinophils in CSU lesions [38]. Furthermore, autoantibodies against the low-affinity IgE receptor (FcεRII: CD23), which can bind the CD23 sequence, which may activate eosinophils, such as major basic protein (MBP) release, are detected in approximately 65% of patients with CSU [39]. The activated eosinophils release MBP and eosinophil peroxidase (EPO), which then induce histamine release from mast cells via MrgX2 [40]. The infiltration of eosinophils into the dermis may also lead to the activation of skin mast cells, either by these eosinophils expressing TF or by the release of MBP and EPO. Basophils do not express TF in response to any stimuli. However, we recently demonstrated that high concentrations of IgE antibody (>1 μM) mildly activate basophils that express IgE-free FcεRI on the cell surface [41]. We also reported that IgE concentrations in patients with CSU were higher compared to healthy donors [41]. Since the lifetime of basophils is as short as 3 days or less, newly differentiated basophils in the bone marrow expressing FcεRIs without IgE are exposed to a high concentration of IgE when these cells move into the blood circulation, resulting in the release of a small amount of histamine and the synergic expression of TF on vascular endothelial cells in patients with CSU [41]. Moreover, basophils express C3a receptor (C3aR) and C5aR, which are increased in CSU skin lesions, and release histamine predominantly in response to C5a [29]. Therefore, basophils may contribute to edema formation by releasing histamine in response to C5a both in the blood circulation and in the dermis. The histamine release from basophils which newly arise from the bone marrow, in response to high concentrations of IgE, may be suppressed by treatment with omalizumab, which promptly neutralizes circulating IgE. The involvement of neutrophils and lymphocytes in CSU was reported and summarized in a recent review article [42].

## 6. The Role of the Complement System in CSU

The complement system is known to play an important role in host defense, which leads to the opsonization and killing of microorganisms, such as bacteria. The complement system consists of a series of several proteins, including complement (C)1–C9 in the blood and tissue fluids [43]. Most of the complement components are normally inactive, but become sequentially activated via enzyme cascades. The activation of one protein enzymatically cleaves and activates the next factor in the cascade, in response to the recognition of molecular components of microorganisms [44]. The cascade of the complement system is classified into three major pathways: the classical pathway, the alternative pathway and the lectin pathway. The classical pathway is initiated by the activation of C1 protein bound to the immune complex of immunoglobulin, whereas the alternative cascade starts with the activation of C3, cleaving it into C3b and C3a (anaphylatoxin), which promotes inflammation. C3b then cleaves C5 into C5b and C5a (anaphylatoxin), which activates macrophages, neutrophils, basophils and also skin mast cells expressing C5aRs. It has been shown that thrombin (FIIa) is capable of generating C5a, a complement activation product, in the absence of C3b [44]. Clark et al. suggested that thrombin and plasmin may contribute to nontraditional complement activation during liver regeneration even in the absence of C4 [45]. In the setting of systemic inflammation, the activation of the coagulation cascade is accompanied by a profound activation of the complement system, resulting in the generation of the anaphylatoxins, C3a and C5a [46]. According to a previous report, C5a also induces TF expression in human endothelial cells and may therefore be involved in the activation of the blood coagulation pathway [47]. Zhu et al. suggested that the plasma level of C5a is increased in patients with CSU [15]. We recently demonstrated that the active forms of coagulation factors (FXa and FIIa) produce C5a from C5 (Figure 2 and Figure 3) [29]. Moreover, C5a, but not C3a, induced the release of histamine from human skin mast cells and peripheral blood basophils via C5aR without antigens and IgE interaction (Figure 3) [29]. Notably, C5aRs are expressed on the surfaces of skin mast cells, but not of other kinds of human mast cells. Although the histamine-release-inducing activity of C3a on human skin mast cells and peripheral blood basophils is low, C3a may regulate other conditions and functions via C3aR expressed on skin mast cells and basophils.

## 7. Mechanism of CSU Induced by the Coagulation–Complement System

The activation of skin mast cells followed by the development of urticaria via the extrinsic coagulation cascade may be explained by the following model. As we have described above, a combination of infections by pathogens, such as Gram-negative bacteria with LPS and histamine, or that of other TF inducers, such as TNF-α, IL-1β, VEGF and IL-33, first induces the synergistic expression of TF on the surfaces of vascular endothelial cells. Mildly activated basophils in CSU patients in response to autoantibodies such as IgGs against FcεRI or IgE antibody, binding of autoantigens, such as IL-24, TPO, TF and/or dsDNA to IgE, and/or high concentrations of IgE (>1 μM) may release a small amount of histamine. TLR agonists, such as LPS, and anti-CD23 autoantibodies also induce TF expression on the surfaces of monocytes and eosinophils, respectively. TF expressed either on vascular endothelial cells, eosinophils or monocytes then activates the extrinsic coagulation cascade, resulting in the production of the active forms of coagulation factors, such as FVIIa, FXa and FIIa. FXa and FIIa increase vascular permeability via PAR-1 expressed on vascular endothelial cells and induce leakage of plasma containing active forms of coagulation factors and complement components (Figure 3). Active forms of coagulation factors, such as FXa and FIIa, may convert C5 in serum to C5a in intravascular and leaked plasma, which then induces histamine release from skin mast cells and basophils via C5aR (Figure 2 and Figure 3). We also reported that a fibrinolysis factor, plasmin, also converts C5 to C5a, resulting in mast cell activation [29]. Finally, a large amount of histamine released from the activated mast cells and recruited basophils in the dermis in response to C5a and/or autoantibodies results in long-lasting and robust edema formation, recognized as wheals, through H1R expressed on vascular endothelial cells (Figure 3). Meanwhile, activated eosinophils migrate outside of blood vessels and release stored inflammatory mediators, such as MBP and EPO, which may also induce or enhance the degranulation of skin mast cells via MrgX2. In addition to the mechanisms of the coagulation–complement system, neuropeptides, such as SP and neuromedin-U, may also activate skin mast cells via upregulated MrgX2 which is upregulated in patients with CSU [48]. Bossi et al. suggested that plasma from patients with CSU causes the release of histamine from human mast cell lines, LAD2 and HMC-1, by an IgE-FcεRI independent mechanism [49]. Moreover, Cugno, et al. reported the presence of a molecule that is smaller than 30 kDa and induces histamine release from mast cells in patients with CSU [50]. These factors in leaked plasma may also contribute to the activation of skin mast cells. C3a produced by the protease activity of FXa and FIIa may regulate skin mast cells and basophils by non-histamine release activity via C3aR expressed on their surface.

## 8. Conclusions

We have shown a possible role of TF-expressing cells, such as vascular endothelial cells, monocytes and eosinophils, extrinsic coagulation cascade/activated coagulation factors (FXa, FIIa) and activated complement (C5a) in the pathogenesis of CSU. Further studies on the relationship between CSU and coagulation–complement systems may allow us to develop useful therapeutic approaches, hopefully low-molecular-weight antagonists of C5a and treatments for patients with severe and refractory CSU, targeting the TF-triggered extrinsic coagulation pathway–complement system–mast cell and/or basophil activation axis.
